# Do white matter hyperintensities mediate the association between brain iron deposition and cognitive abilities in older people?

**DOI:** 10.1111/ene.13006

**Published:** 2016-04-20

**Authors:** M. Valdés Hernández, M. Allerhand, A. Glatz, L. Clayson, S. Muñoz Maniega, A. Gow, N. Royle, M. Bastin, J. Starr, I. Deary, J. Wardlaw

**Affiliations:** ^1^ Department of Neuroimaging SciencesCentre for Clinical Brain SciencesUniversity of EdinburghEdinburghUK; ^2^ Centre for Cognitive Ageing and Cognitive EpidemiologyUniversity of EdinburghEdinburghUK; ^3^ College of Medicine and Veterinary MedicineUniversity of EdinburghEdinburghUK; ^4^ Department of PsychologySchool of Life SciencesHeriot‐Watt UniversityEdinburghUK

**Keywords:** ageing, cognition, iron deposits, MRI, white matter hyperintensities

## Abstract

**Background and purpose:**

Several studies have reported associations between brain iron deposits (IDs), white matter hyperintensities (WMHs) and cognitive ability in older individuals. Whether the association between brain IDs and cognitive abilities in older people is mediated by or independent of total brain tissue damage represented by WMHs visible on structural magnetic resonance imaging (MRI) was examined.

**Methods:**

Data from 676 community‐dwelling individuals from the Lothian Birth Cohort 1936, with Mini‐Mental State Examination scores >24, who underwent detailed cognitive testing and multimodal brain MRI at mean age 72.7 years were analysed. Brain IDs were assessed automatically following manual editing. WMHs were assessed semi‐automatically. Brain microbleeds were visually counted. Structural equation modelling was used to test for mediation.

**Results:**

Overall, 72.8% of the sample had IDs with a median total volume of 0.040 ml (i.e. 0.004% of the total brain volume). The total volume of IDs, significantly and negatively associated with general cognitive function (standardized *β* = −0.17, *P* < 0.01), was significantly and positively associated with WMH volume (std *β* = 0.13, *P* = 0.03). WMH volume had a significant negative association with general cognitive function, independent of IDs (std *β* = −0.13, *P* < 0.01). The association between cognition and IDs in the brain stem (and minimally the total brain iron load) was partially and significantly mediated by WMH volume (*P* = 0.03).

**Conclusions:**

The negative association between brain IDs and cognitive ability in the elderly is partially mediated by WMHs, with this mediation mainly arising from the iron deposition load in the brain stem. IDs might be an indicator of small vessel disease that predisposes to white matter damage, affecting the neuronal networks underlying higher cognitive functioning.

## Introduction

As people age, iron accumulates in several brain regions and cell types 1, 2.This accumulation, specifically in the corpus striatum and substantia nigra, is reported to be associated with cognitive decline and neurodegeneration 3, 4.

**Figure 1:**
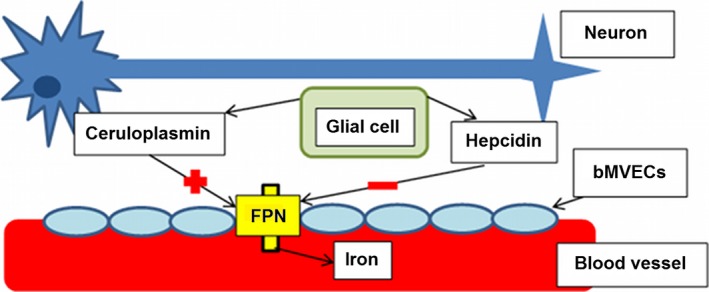
Schematic representation of brain iron regulation mechanisms as detailed in McCarthy and Kosman 5 (FPN, ferroportin; bMVECs, brain microvascular endothelial cells). Celuroplasmin, released from glial cells, promotes the removal of iron from the brain via ferroportin, an exporter located in the microvascular endothelial cells, with hepcidin being a negative regulator of ferroportin. Glial fibrillary acidic protein‐positive cells express hepcidin. Hepcidin binds to and induces ubiquitination of FPN triggering FPN internalization and degradation. By this mechanism, the interaction of hepcidin with ferroportin regulates the flow of iron into plasma, and thereby regulates the iron distribution.

**Figure 2:**
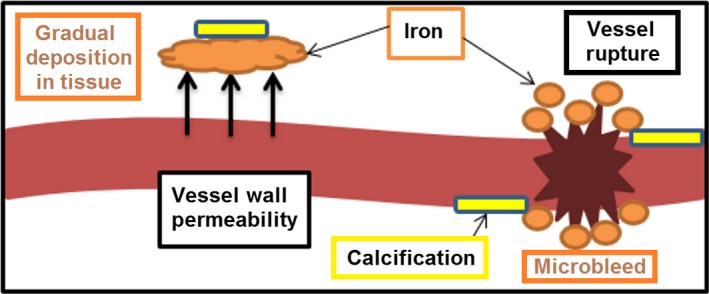
Schematic representation of the types and sources of mineral deposits found in brains of older adults: gradual iron deposition in tissue and brain microbleeds mainly determined by dysfunction in the permeability of the vessel wall and vessel rupture respectively; and calcifications.

Iron may accumulate in tissue via dysfunctional brain iron regulatory mechanisms 5. Changes in glial cell function could alter the exportation of iron leading to its accumulation in tissue (Fig. 1), which, if prominent, can be identifiable in structural magnetic resonance imaging (MRI). In addition, over time, very small chronic haemorrhages known as microbleeds originating in abnormal blood vessels (i.e. capillaries) produce paramagnetic haemosiderin 6. Glial cells, if dysfunctional, can fail to clear these haemosiderin micropools from tissue resulting in their accumulation. Specifically, these are radiologically identified as round hypointensities of less than 10 mm diameter on T2*‐weighted gradient echo MRI. The aggregation of iron deposits (IDs) − coming from the two sources mentioned − with other minerals, one of which is calcium, produces so called ‘calcifications’, thought to prevent the toxic effects of iron on brain tissue 7. It is important to note that, in comparison with microbleeds, mineral deposition occurs more gradually and is related to the permeability of vessel walls in addition to glial cell function. Calcifications, however, may have other sources as yet unidentified (Fig. 2). As the build‐up of iron leads to oxidative stress when ferrous iron interacts with hydrogen peroxide forming reactive hydroxyl radicals 8, it is hypothesized that oxidative stress caused by metal accumulation in brain tissues may be one of many factors associated with neurodegeneration and cognitive decline.

White matter hyperintensities (WMHs), observed in the white matter and subcortical grey matter on fluid attenuation inversion recovery (FLAIR) and T2‐weighted structural MRI, are also common in brains from older adults, and have been associated with both neurodegeneration 9 and cognitive decline 10. Their total volume has been associated with the load of iron deposition in the globus pallidus 11, where this deposition is predominant in older individuals 12. Despite reports that both WMHs and iron deposition progress gradually with age 13, WMHs appear generally earlier and their progression is more dynamic and associated with processes such as hypertension 14, not seeming to be associated with IDs. However, there is also evidence linking the overall WMH progression with endothelial 15 and blood−brain barrier permeability dysfunction 16, also related to the formation of IDs (Figs 1 and 2).

The iron accumulation associated with ageing is known to occur primarily in ferritin‐rich areas associated with motor activity, hypothesized to have unknown cell types that protect against demyelination 17, which are programmed to store iron creating a reservoir to the central nervous system. Consequently, as iron‐rich pools are located between the region of uptake rich in blood vessels and the region where iron is demanded for myelin production 18 it is expected that WMHs and brain iron accumulation do not share the same anatomical regions, and it is theorized that WMHs cause a reduced demand for iron 18, accentuating cognitive decline (less soluble iron leading to poorer cognition) as their load increases with age.

Given the evidence for the common co‐occurrence of WMHs and IDs 19, their common neurological substrates and effect in cognition, and the characteristics of their progression, it was hypothesized that WMHs may accelerate or accentuate the known effect that IDs have on cognition 19. If this is the case, it is predicted that (i) IDs will be associated with increased WMH load and (ii) WMH load will partially explain any association between IDs and cognition. Given the low likelihood of WMHs and brain iron deposition being in the same anatomical region and evidence on the interaction of the different mechanisms that are known to lead to both brain iron accumulation in normal ageing and WMHs 20, 21, their spatial distribution was also analysed hypothesizing that any association between WMHs and IDs may be due to a systemic and not a localized effect.

These hypotheses were investigated in a large narrow‐age cohort of people in their 70s. From this cohort, studies independently relating WMHs and IDs with cognition 3, 10, 19 and with other related factors (e.g. nutritional 22 or vascular risk 19, 23) have been published (http://www.lothianbirthcohort.ed.ac.uk/). As calcifications do not necessarily share the same substrate and effect as IDs, they are not considered in the analysis.

## Materials and methods

**Figure 3:**
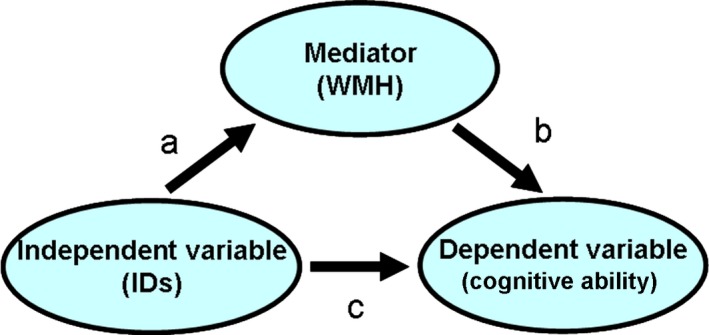
The three‐variable non‐recursive causal model to explore the mediating role of white matter hyperintensities (WMHs) on the effect that brain iron deposits(IDs) have on cognition in the elderly. Path a explores whether variations in the amount of IDs account for variations in the WMH load, path b explores whether variations in the WMH load account for variations in the cognitive outcomes, and path c explores the direct effect of IDs on cognition.

Structural brain MRI and cognitive data from 676 community‐dwelling individuals from the Lothian Birth Cohort 1936 at mean age 72.7 years (SD 0.7, range 71.1–74.3) from whom written informed consent was obtained under protocols approved by the Lothian (REC 07/MRE00/58) and Scottish Multicentre (MREC/01/0/56) Research Ethics Committees were analysed. From the MRI, brain IDs were assessed automatically following manual editing and WMHs were assessed semi‐automatically. Brain microbleeds were visually counted. The cognitive variables analysed were fluid intelligence (g), general processing speed (g‐speed) and general memory (g‐memory). They were generated using principal component analysis from batteries of well‐validated cognitive tests 3. The structural equation modelling shown in Fig. 3 was used to test for mediation. Details of the imaging protocol, cognitive tests and statistical analyses can be found in Data S1.

## Results

### Sample characteristics

The descriptive statistics of the imaging and cognitive variables involved are given in Table 1. IDs appeared mainly in the corpus striatum and brainstem. In the corpus striatum, IDs were identified in 477 participants (70.8% of the sample), in the brainstem in 87 participants (12.9%) and elsewhere in 51 participants (7.6%). In general, IDs were present in 72.8% of the sample (490 participants). The median total volume of IDs in the whole sample was 0.04 ml (0.003% of the intracranial volume (ICV)), and the median load in those subjects who had them was 0.1 ml (0.007% of the ICV) (interquartile range 0.25 ml), ranging from 0.002 ml to 3.22 ml. From the 51 individuals who had microbleeds identified as ‘certain’ 24, 37 (72.5%) had only one microbleed. In general, 22 microbleeds were identified in the basal ganglia, four in the brainstem and 75 elsewhere. WMH burden in this cohort has been characterized in detail previously 10, 25. They were absent in only 7/655 individuals. The WMH median volume in this sample was 7.7 ml (0.53% of the ICV) (interquartile range 13.35 ml) (Table 1).

**Table 1:** Imaging and cognitive variables considered in the analyses

Parameter	Mean			Standard deviation		
	Men	Women	Total	Men	Women	Total
General cognition (i.e. g)	−0.020	0.054	0.014	1.074	0.927	1.007
Processing speed (i.e. g‐speed)	−0.047	0.058	0.003	1.064	0.964	1.018
General memory (i.e. g‐memory)	−0.094	0.109	0.003	1.073	0.950	1.020
Brain tissue volume (ml)	1173.869	1068.147	1123.980	98.396	86.472	106.858
Intracranial volume (ml)	1535.940	1355.658	1450.977	113.098	102.006	140.571
	Median			Interquartile range		
Volume of IDs in the corpus striatum (ml)^a^	0.082	0.094	0.088	0.224	0.222	0.224
Volume of IDs in the brain stem (ml)^a^	0.086	0.102	0.091	0.111	0.123	0.120
Volume of IDs elsewhere (ml)^a^	0.022	0.028	0.026	0.122	0.113	0.120
Total volume of IDs (ml)	0.046	0.036	0.040	0.201	0.187	0.196
WMH volume (ml)	7.870	7.402	7.704	13.218	13.866	13.348
	Incidence (i.e. number of participants with IDs in the region)			Volume range (ml)		
Corpus striatum IDs	260/358	217/316	477/674	2.768	2.670	(0 – 2.768)
Brain stem IDs	61/358	26/316	87/674	0.748	0.554	(0–0.748)
IDs elsewhere	29/358	22/316	51/674	1.404	0.946	(0–1.404)
Brain region	Number of ‘certain’ microbleeds per region in the sample			Frequency of occurrence (1/more than 1 microbleed) in the sample		
Basal ganglia	15	7	22	6/2	5/1	11/3
Brain stem	2	2	4	2/0	0/1	2/1
Elsewhere	55	20	75	16/6	8/4	24/10

IDs, iron deposits; WMHs, white matter hyperintensities. Mean and standard deviation are given for variables normally distributed across the sample. For variables not normally distributed median and interquartile range are given instead. ^a^Considers only participants who had the parameter (i.e. regional iron deposition) measured.

### Spatial distribution of WMHs and IDs

White matter hyperintensities were distributed symmetrically between right and left sides of the brain in periventricular, centrum semiovale and external capsule white matter, with a greater load in the frontal periventricular regions as opposed to the occipital regions. Very few WMHs were identified in the corpus striatum. The spatial distribution and probability of occurrence of WMHs was consistent with distribution patterns shown by other studies of ageing 26 and with that of the subsample that did not have stroke analysed elsewhere 27.

**Figure 4:**
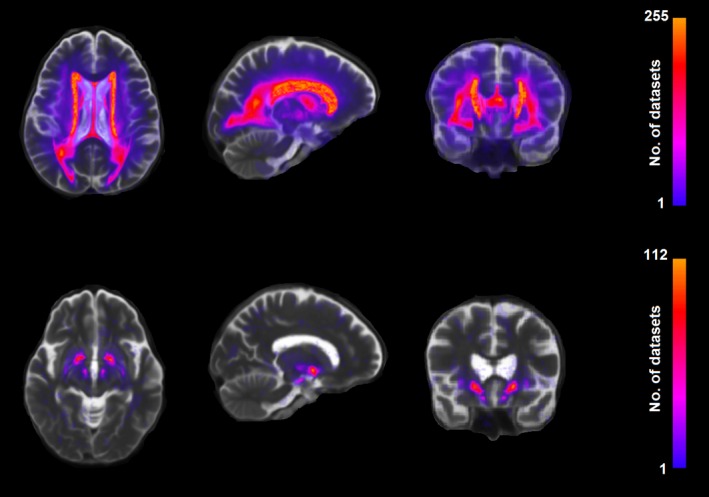
Maximum intensity projections of standard space probability distribution maps in the (from left to right) axial, sagittal and coronal views of WMHs (first row) and IDs (second row) in the sample.

In contrast, the highest load of IDs was in the globus pallidus (Fig. 4), extending to the putamen in cases with prominent mineralization 12 and in three cases of old haemorrhages, followed by the brainstem. The anatomical distribution pattern of IDs was consistent with those reported by histopathological studies 28, 29. Figure 4 shows a comparative standard space distributional map of the occurrence of IDs and WMHs in the sample.

### Bivariate associations of IDs with WMH volume and cognition (paths a and c in the mediation model)

Total volume of brain IDs was significantly and negatively associated with all indicators of general cognitive abilities at mean age 72.7 years (Table 2, path c). Associations of total and corpus striatum volumes of IDs with general cognition (*β* = −0.17 and −0.14 respectively), processing speed (*β* = −0.13 and −0.10 respectively) and general memory (*β* = −0.13 and −0.10 respectively) were significant (*P* < 0.05) with the exception of the association between volume of IDs in the corpus striatum and processing speed (*P* = 0.06; Table 2, path c). The brainstem volume of IDs was significantly associated with total WMH volume (*β* = 0.14 respectively, *P* = 0.006; Table 2, path a). However, the volume of IDs in the corpus striatum was not significantly associated with WMH volume despite being the main contributor to the total iron deposition load. The total volume of IDs was marginally associated with WMHs when this was adjusted for brain tissue volume (*β* = 0.13, *P* = 0.047; Table 2, path a), but when WMHs and IDs volumes were adjusted for head size (i.e. ICV), this association was not significant.

**Table 2:** Results of the mediation analysis: path a explores whether variations in the volume of iron deposits (IDs) (corrected for brain size) account for variations in the WMH load, path b explores whether variations in the WMH load account for variations in the cognitive outcomes, path c explores the direct effect of iron deposition on cognition and path ab explores the indirect effect of iron deposition on cognition mediated by WMHs (see Fig. 1)

Regional and total volumes of iron deposition (independent variable)	Cognitive parameters (dependent variable)	Path a (associations between volumes of IDs and WMHs) (*β*;* P*)	Path b (associations between WMHs and cognitive abilities) (*β*; p)	Path c (direct effect of iron deposition on cognitive abilities) (*β*;* P*)	Path ab (indirect effect of iron deposition on cognitive abilities, i.e. mediated by WMHs) (*β*;* P*)
Corpus striatum	g	0.091; 0.100	−0.138; 0.004	−0.137; 0.006	−0.012; 0.133
	g‐speed	0.091; 0.100	−0.181; <0.001	−0.096; 0.057	−0.016; 0.110
	g‐memory	0.090; 0.100	−0.092; 0.029	−0.104; 0.033	−0.008; 0.190
Brain stem	g	0.136; 0.006	−0.141; 0.003	−0.067; 0.148	−0.019; 0.028
	g‐speed	0.136; 0.006	−0.180; <0.001	−0.068; 0.145	−0.024; 0.010
	g‐memory	0.136; 0.006	−0.090; 0.035	−0.082; 0.069	−0.012; 0.085
Total	g	0.130; 0.047	−0.128; 0.008	−0.166; 0.003	−0.017; 0.075
	g‐speed	0.130; 0.047	−0.173; <0.001	−0.128; 0.030	−0.022; 0.046
	g‐memory	0.130; 0.047	−0.085; 0.044	−0.127; 0.017	−0.011; 0.143

The sample size for this analysis was *n* = 597, restricted to individuals with usable cognitive and brain imaging data on white matter hyperintensities (WMHs), brain tissue volume and iron deposition.

### White matter hyperintensities as a mediator between IDs and cognition in late adulthood (path ab on the mediation model)

White matter hyperintensities significantly mediated the association of IDs in the brainstem with general cognitive ability [*β* = −0.019, 95% confidence interval (−0.036–0.002), *P* = 0.028] and processing speed [*β* = −0.024, 95% confidence interval (−0.043–0.006), *P* = 0.010] (only the indirect path was significant). Also, WMHs partially and significantly mediated the association between the total amount of IDs and processing speed (both direct paths and indirect paths were significant; Table 2).

## Discussion

### Associations between brain IDs, cognition and WMHs

The negative and significant association between total volume of IDs and cognitive measures in this cohort was reported previously 19. In addition, our results show a negative and significant association between the corpus striatum volumes of IDs and these cognitive measures. This finding is consistent with results from a study on 10 healthy elderly subjects that used quantitative estimates of regional iron 30.

The association between total volumes of IDs and WMHs merits cautious analysis. Histochemical studies have shown that abnormal brain IDs are involved in the pathogenesis of demyelinating diseases 31. WMHs are involved in tissue rarefaction associated with myelin and axon loss, with mild gliosis likely to be associated with the phagocytosis of myelin breakdown products 32.Therefore, it would be expected that both WMHs and total iron deposition volumes would be associated. Their volumes in brain tissue were associated but only with marginal significance. After adjusting for head size this was no longer the case, perhaps driven by the influence that iron deposition in the corpus striatum has on the overall brain iron load. Despite the corpus striatum being highly vascularized, the volume of IDs in this region was not associated with the total volume of WMHs mainly of vascular origin 33. The probability density distribution of WMHs in the corpus striatum, where iron deposition was identified, was low. Conversely, IDs were not found in periventricular regions, where WMHs prevailed. A study concluded that only T2*‐weighted hypointensities in the caudate nucleus showed an association with total WMH volume and other markers of neurodegeneration 18. Blood−brain barrier permeability analyses would be beneficial to disentangle the relationship between these two imaging biomarkers in more detail.

### Mediation effect of WMHs on the association between IDs and cognitive measures

The fact that the association of the total burden of IDs in cognition was partially mediated by WMH volume may indicate that, in general, IDs might be an indicator of small vessel disease that predisposes to white matter damage, thereby affecting the neuronal networks underlying higher cognitive functioning. Special attention should be given to IDs in the corpus striatum, not associated with WMH volume, and which determine the brain iron deposition burden in this cohort. Although it has been suggested 3, 30 that IDs have an effect on reducing processing speed in older age, our results suggest that this effect is neither direct nor mediated by WMHs. The mechanisms through which corpus striatum IDs influence processing speed are therefore worth exploring further.

The mediation effect of WMHs on the association between iron deposition load and cognitive measures was mainly determined by the volume of the regions where this mineral was visibly accumulated in the brain stem (mainly lower midbrain and upper brain stem as Fig. 4 shows). Studies have shown that ferroportin plays a key role in iron regulation 20, 34. This transmembrane protein is widely expressed in the deep cerebellar nuclei, brain stem, endothelial cells of the blood−brain barrier (Fig. 1) and presynaptic vesicles. Its disrupted expression or function can increase the iron concentration in the brain. The presence of free iron in synapses could potentially expose the synaptic membranes to iron‐dependent oxidation and damage, implying that abnormalities in iron homeostasis may have a direct adverse effect on synaptic integrity and, consequently, be a precursor of WMHs. The mechanisms that underlie the mediation role of WMHs on the effect that iron accumulation mainly in the brain stem has on cognition or whether this mediation is apparent, driven by co‐localized disturbances in the proteins involved in the uptake, release, storage, intracellular metabolism and regulation of iron in the brain, requires further investigation.

### Strengths and limitations

Strengths of this paper are the analysis of the role that WMHs play in the effect IDs have on cognition, explored here for the first time, the use of several recognized measures of cognitive ability on a large cohort of community‐dwelling older individuals, and the use of robust quantitative methods to measure IDs and WMH volumes. Given that a birth cohort was analysed, the influence of age in the iron accumulation process could not be explored. However, as our sample was representative of the ID distribution and WMH load of community‐dwelling septuagenarian Caucasian individuals, this study is relevant for epidemiological and ageing studies.

As the actual volume of iron accumulation in tissues cannot be accurately determined using structural MRI techniques 19, our analyses are based on volumetric measurements that, although accurate, rather reflect the effect that iron particles in brain tissue have on the MR signal. This effect is partly affected by the susceptibility of the metal/metalloid particles influenced by their aggregation, proportion and interaction with the underlying tissue amongst other factors 12 and merits more research. Due to the low incidence of microbleeds in this cohort they were not analysed separately. The replication of our analyses on cohorts with higher prevalence of this type of iron deposition is therefore a necessity.

Although the total iron measures obtained may be influenced by biologically inert forms as part of ferritin, hemosiderin and other macromolecules, they might be predictive for bioactive iron compounds and, as such, have relevance for studies of ageing 2. The fact that in our normal ageing cohort these IDs exist in volume and count in the same regions as they accumulate in neurodegenerative diseases 17 constitutes further evidence that ageing and some neurodegenerative diseases share similar mechanisms involving iron. A longitudinal study of the association of the two imaging markers studied here between themselves and with cognition will help in understanding the mechanisms that are known to contribute to cognitive decline and neurodegeneration in late adulthood.

## Disclosure of conflicts of Interest

The authors declare no financial or other conflicts of interest.

## Supporting information


**Data S1.** Online methods
