# Supplementary material for: Do white matter hyperintensities mediate the association between brain iron deposition and cognitive abilities in older people?
Source: Eur J Neurol. 2016 Apr 20;23(7):1202–9. doi: 10.1111/ene.13006 (PMC4950475; doi:10.1111/ene.13006)
Supplement: Supplementary file 1 — Data S1. Online methods [file ENE-23-1202-s001.docx]

**Do white matter hyperintensities mediate the association between brain iron deposition and cognitive abilities in older people?**

**Online Methods**

**Subjects**

The Lothian Birth Cohort 1936 (LBC1936) comprises mostly healthy community-dwelling older people. Most participants were living in the Edinburgh area of Scotland in older age. All were born in 1936 and most took an intelligence test at age 11 years in the Scottish Mental Survey of 1947[1]. They were about mean age 70 when 1091 individuals were recruited to a study of healthy cognitive ageing; of these, 700 participants (328 females and 372 males) had a brain MRI scan at mean age 72.7 years (SD 0.7, range 71.1 to 74.3). From the 700 brain image sets, 676 had the relevant sequences to assess brain iron deposition and WMH. Written informed consent was obtained from all participants under protocols approved by the Lothian (REC 07/MRE00/58) and Scottish Multicentre (MREC/01/0/56) Research Ethics Committees.

**MRI scans**

MRI scans were acquired using a 1.5T GE Signa Horizon HDxt clinical scanner (General Electric, Milwaukee, WI, USA) operating in research mode using a self-shielding gradient set with maximum gradient of 33 mT/m and an 8-channel phased-array head coil. The imaging protocol is fully described elsewhere[2]. For this particular study, we used data obtained from processing coronal T1-weighted (T1W) volumes acquired with a 3D inversion recovery prepared fast gradient echo sequence (TR/TE/TI = 9.7/3.984/500 ms, flip angle α = 8°, bandwidth 15.63 kHz, voxel size 1x1x1.3 mm^3^), axial FLAIR volumes (TR/TE/TI = 9000/140/2200 ms, bandwith15.63 kHz, voxel size 1x1x4 mm^3^), and axial T2*W volumes acquired with a 2D gradient-echo sequence (GRASS, TE/TR = 15/940 ms, flip angle α = 20°, bandwidth 12.5kHz, voxel size 1x1x2 mm^3^). None of these sequences had interslice gap and, for all, the field-of-view (FOV) in the acquisition plane was 256x256 mm^2^.

**Image processing**

Multifocal T2*W hypointensities in the corpus striatum were assessed fully automatically using the method described in Glatz et al. (2014)[3], and freely available at <https://github.com/aglatz/mineral-deposit-segmentation-pipeline/tree/master/libBRIC/mineral-deposit-segmentation>. T2*W hypointensities were separately identified, extracted and quantified in the pre-processed T2*W scans semi-automatically using the ‘Object Counter’ module in Analyze^TM^ 10.0 following a validated procedure[4,5]. Brain microbleeds were visually assessed by an experienced neuroradiologist using the Brain Observer Micro Bleed Scale (BOMBS)[6]. Intracranial volume (ICV) and WMH volume were segmented as described in the study protocol[2] using MCMxxxVI_ALE, a software tool freely available from ([www.sourceforge.net/projects/bric1936](http://www.sourceforge.net/projects/bric1936)). All measurements were visually checked and manually rectified if needed.

**Cognitive testing and cognitive variables**

For this work, we used cognitive measures obtained at the time of MRI scanning (mean age 72.7, SD 0.7 years). These cognitive variables[1] were: the general cognitive components of fluid intelligence (g), general processing speed (g-speed) and general memory (g-memory). These general cognitive ability measures were generated using principal component analysis from batteries of well-validated cognitive tests as described in [7]. To derive g, six subtests of the WAIS-III^UK[8]^ were used: Digit Symbol, Digit Span Backward, Symbol Search, Letter-Number Sequencing, Block Design and Matrix Reasoning. g-memory was derived from five subtests from the WMS-III^UK[9]^: Logical Memory Total Immediate and Delayed Recall, Verbal Paired Associates Immediate and Delayed Recall, and Spatial Span Total Score; and two subtests from the WAIS-III^UK^: Letter-Number Sequencing and Digit Span Backward. g-speed was obtained from two reaction time tests (Simple Reaction Time and Choice Reaction Time), an Inspection Time test, and two WAIS-III^UK^ subtests: Digit Symbol and Symbol Search.

**Statistical analyses**

The total and regional iron and WMH volumes were standardised by ICV and brain tissue volume to account for inter-individual differences in head size with and without the confounding effect of global brain atrophy. Age in days at the time of MRI scanning and/or cognitive testing was used as a covariate in all models. To test for a possible mediating role of WMH in the association between iron deposits and each cognitive domain, we used the procedure described by Baron and Kenny (1986)[10]. In this analysis, performed in Mplus 6.1, the putative mediator variable was the percentage of WMH in ICV or brain tissue volume, the dependent variables were the cognitive variables and the independent variables were the regional (and total) iron deposits (Figure 3 in the main test).

**Spatial distribution analyses**

To generate spatial probability distribution maps of WMH and IDs, we first linearly aligned the volumes used as the base for the segmentations (i.e. T2*W for IDs and T2W for WMH) to a cohort-specific brain template in standard space using FSL-FLIRT[11]. Then, we non-linearly transformed the binary masks of WMH and IDs to the cohort template using the linear transformation matrix as input to the tool NiftyReg[12] (<http://sourceforge.net/projects/niftyreg/>) within the TractoR project[13] (<http://www.tractor-mri.org.uk/registration>). Lastly, the warped binary masks were added to generate the spatial probability distribution maps of WMH and IDs.

Reference List

[1] Deary,IJ, Gow,AJ, Taylor,MD, Corley,J, Brett,C, Wilson,V et al. The Lothian Birth Cohort 1936: a study to examine influences on cognitive ageing from age 11 to age 70 and beyond. *BMC Geriatr* 2007; **7**: 28.

[2] Wardlaw,JM, Bastin,ME, Valdes Hernandez,MC, Munoz Maniega,S, Royle,NA, Morris,Z et al. Brain aging, cognition in youth and old age and vascular disease in the Lothian Birth Cohort 1936: rationale, design and methodology of the imaging protocol. *Int J Stroke* 2011; **6**: 547-559.

[3] Glatz,A, Bastin,ME, Kiker,AJ, Deary,IJ, Wardlaw,JM, and Valdes Hernandez,MC. Automated segmentation of multifocal basal ganglia T2*-weighted MRI hypointensities. *Neuroimage* 2015; **105**: 332-346.

[4] Valdes Hernandez,MC, Jeong,TH, Murray,C, Bastin,ME, Chappell,FM, Deary,IJ et al. Reliability of two techniques for assessing cerebral iron deposits from structural MRI. *J Magn Reson Imaging* 2011; **33**: 54-61.

[5] Valdes Hernandez,MC, Glatz,A, Kiker,AJ, Dickie,DA, Aribisala,BS, Royle,NA et al. Differentiation of calcified regions and iron deposits in the ageing brain on conventional structural MR images. *J Magn Reson Imaging* 2014; **40**: 324-333.

[6] Cordonnier,C, Potter,GM, Jackson,CA, Doubal,F, Keir,S, Sudlow,CLM et al. Improving interrater agreement about brain microbleeds. Development of the Brain Observer MicroBleed Scales (BOMBS). *Stroke* 2009; **49**: 94-99.

[7] Penke,L, Valdes Hernandez,MC, Muñoz Maniega,S, Gow,AJ, Murray,C, Starr,JM et al. Brain iron deposits are associated with general cognitive ability and cognitive aging. *Neurobiol Aging* 2012; **33**: 510-551.

[8] Wechsler,D. WAIS-III^UK^ administration and scoring manual. 1998;

[9] Wechsler,D. WMS-III^UK^ administration and scoring manual. 1998;

[10] Baron,RM and Kenny,DA. Moderator-mediator variables distinction in social psychological research: conceptual, strategic and statistical considerations. *J Personal Soc Psychol* 1986; **51**: 1173-1182.

[11] Jenkinson,M, Bannister,P, Brady,M, and Smith,S. Improved optimization for the robust and accurate linear registration and motion correction of brain images. *Neuroimage* 2002; **17**: 825-841.

[12] Modat,M, Ridgway,GR, Taylor,ZA, Lehmann,M, Barnes,J, Hawkes,DJ et al. Fast free-form deformation using graphics processing units. *Comput Methods Programs Biomed* 2010; **98**: 278-284.

[13] Clayden,JD, Munoz Maniega,S, Storkey,AJ, King,MD, Bastin,ME, and Clark,CA. TractoR: magnetic resonance imaging and tractography with R. *J Stat Softw* 2011; **44**: 1-18.
